# Intussusception Caused by Cecal Duplication in an Adult: A Case Report

**DOI:** 10.1155/2022/9520191

**Published:** 2022-10-10

**Authors:** Eric Bergeron, Normand Gervais

**Affiliations:** ^1^Department of Surgery, Charles-LeMoyne Hospital, Greenfield Park, Canada; ^2^Department of Surgery, Centre Hospitalier Régional du Grand-Portage, Rivière-du-Loup, Canada

## Abstract

Cecal duplication is a rare congenital malformation and majority of the cases are discovered in the first years of life. Ileocolic intussusception is also a rare situation encountered in adults. A 19-year-old female presented with acute abdominal pain and bowel occlusion in relation with an ileocecal intussusception. She underwent an emergent laparotomy and ileocecal resection. A cecal duplication cyst was found to be the cause of the intussusception. While duplications and intussusception are very rare situations encountered in the adult life, the presence of both at the same time remains frankly anecdotal. The present case demonstrates that intussusception may likely be involved with any cecal lesion, like duplication.

## 1. Introduction

Alimentary tract duplication is a rare congenital malformation [1]. It is estimated to occur in about 1/10000 live births [1, 2]. More than 80% of these cases are observed before the age of 2 years [3]. A very low proportion of patients attain adulthood with a “silent” duplication [4]. Colonic duplication occurs in 7% to 15% of cases [4–6] with the cecum being the rarest site [3]. Preoperative diagnosis is very unusual, and unless there is an acute abdominal situation, the duplication may remain unsuspected [1, 2, 4, 7].

We present a case of cecal duplication causing an intussusception in a young adult female. This is, to our knowledge, only the third reported case of cecal duplication, with such an unusual presentation in an adult [3, 5].

## 2. Case Presentation

**Figure 1:**
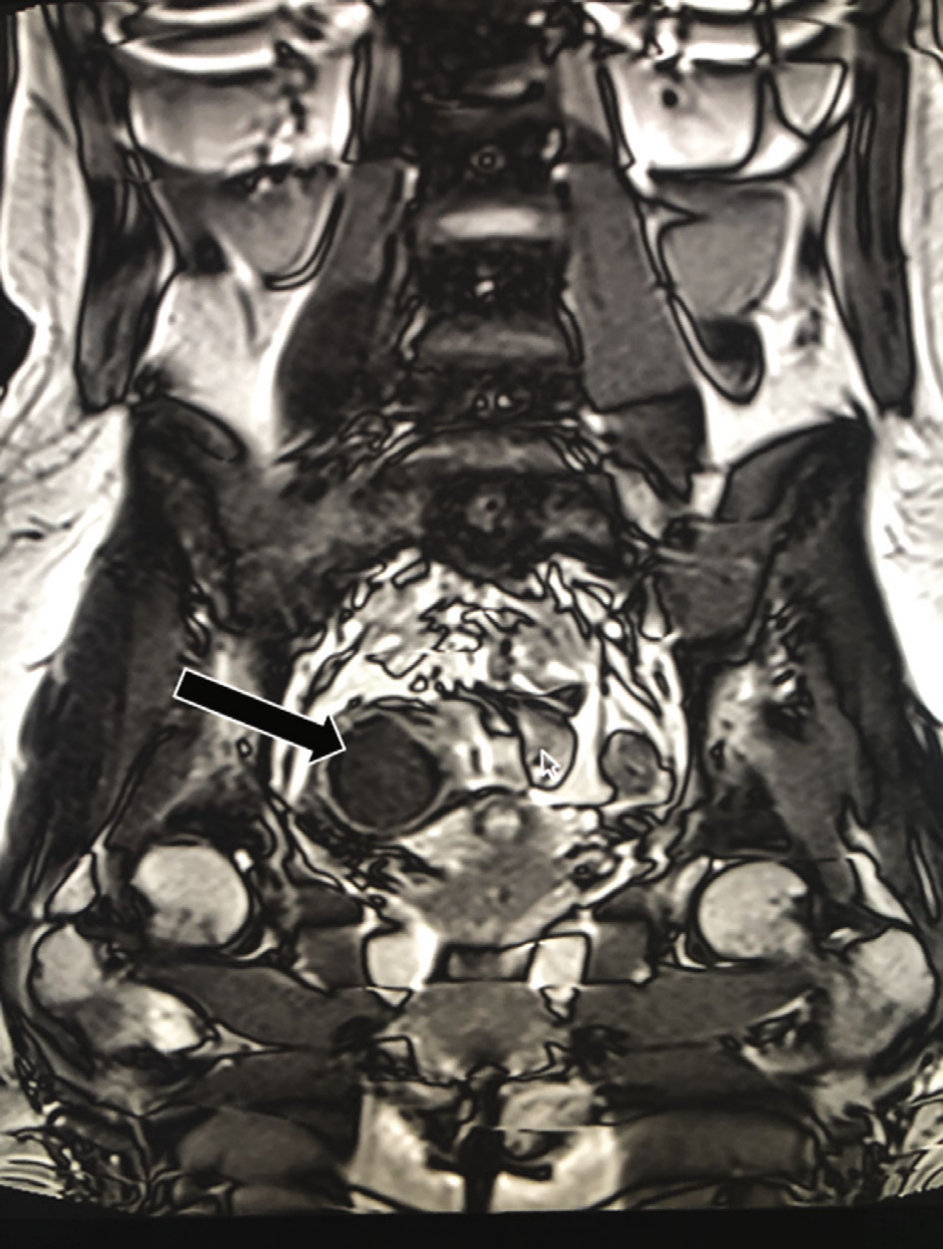
Magnetic resonance imaging showing a cyst at the right iliac fossa.

A 19-year-old Caucasian female without significant past medical history presented at emergency room for abdominal pain. The pain was situated in the right iliac fossa and was crampy in nature. She was sexually active. She denied nausea, vomiting, diarrhea, bloody stools, or weight loss. She was not febrile. The patient appeared in good shape. Abdominal examination showed no distension, no defense, and no rebound tenderness. A light sensitivity was provoked, and the possibility of a mass was felt in this thin patient. White cell count was normal. Ultrasound showed a 69 mm × 33 mm × 28 mm cystic mass in the right parauterine region. There was a distinct 41 mm ovarian cyst. The cystic mass was compared with an ultrasound done nine months before. She then consulted her family doctor for crampy abdominal pain. She also had a magnetic resonance imaging showing a cystic mass measuring 65 mm × 32 mm × 30 mm (Figure 1). This mass was adjacent to the uterus and is in contact with the small bowel, but distinct from it and from the ovary. The presumed diagnosis was a right hydrosalpinx. She denied any recurrence of significant pain between both episodes. With present clinical picture and ultrasound, along with no evident progression between episodes, presumed diagnosis remained a right hydrosalpinx but the possibility of an appendiceal mucocele was now evoked.

The patient was scheduled to undergo a shortly planned laparoscopic intervention. However, she consulted at the emergency room three days after the initial visit for increased abdominal pain. Again, the physical exam and laboratory testing were not conclusive. It was decided to undergo immediate laparoscopy. During the intervention, no hydrosalpinx was noticed. There was a small right ovarian cyst, appearing benign. A serous liquid was aspirated. The right colon, the appendix, the small intestine, and the mesentery appeared normal. With palpation forceps, no mass could be felt. Appendectomy was carried out, and the operation was terminated. The patient was discharged the day after. A colonoscopy was further scheduled to investigate the colon and terminal ileum.

**Figure 2:**
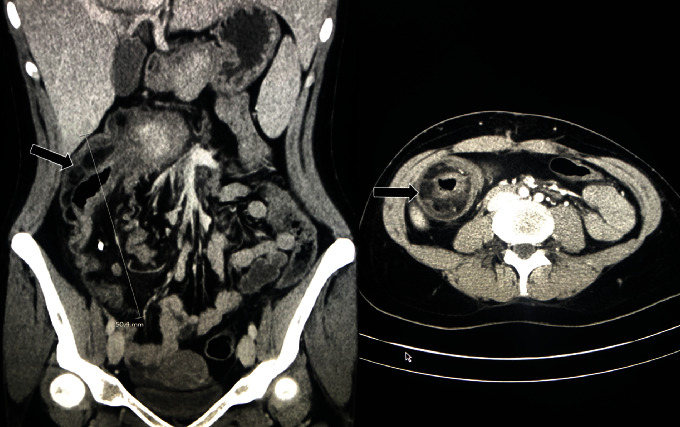
Abdominal computed tomography showing the ileocecal intussusception (left: coronal; right: transverse).

**Figure 3:**
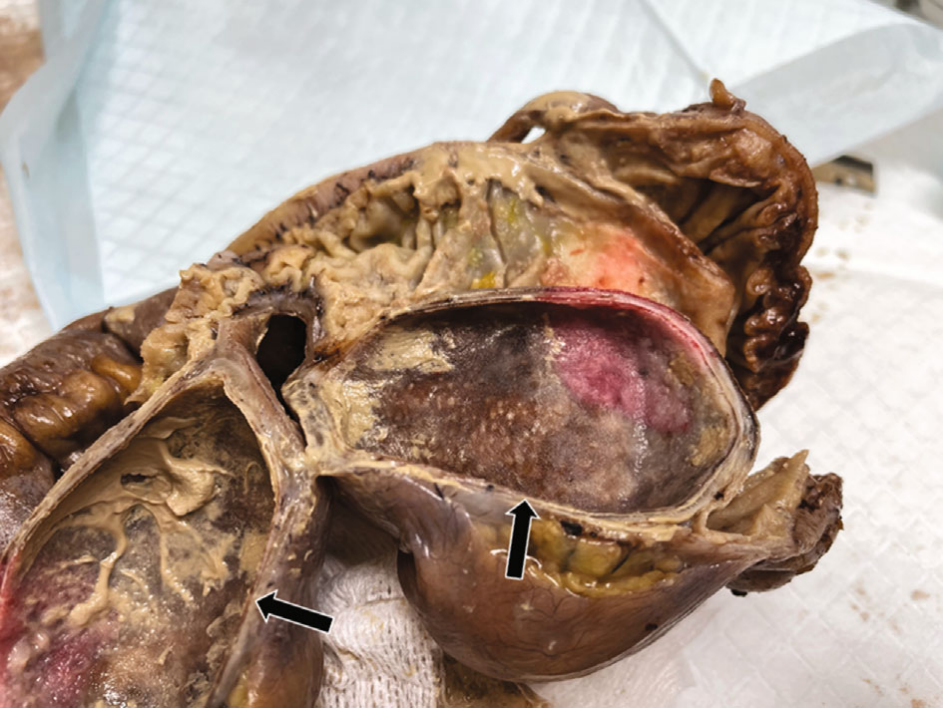
Surgical resection showing the cecal replication cyst after formaldehyde fixation.

Thirteen days later, the patient presented to the emergency room, this time in severe abdominal pain, abdominal distension and vomiting. Her temperature was 37.5°C. White cell count was 15.2 × 10^9^/L (Normal: 5.0-12.0). CT-scan showed ileocecal intussusception (Figure 2). She was prompted to the operating room. She underwent an ileocecal resection with primary anastomosis through a midline incision. The patient recovered uneventfully. The examination of the pathologic specimen showed a cecal duplication that caused the intussusception (Figure 3).

## 3. Discussion

Colonic duplication is very rarely observed in adulthood [3]. With an estimated incidence of alimentary tract duplication of 1/10000 live births [1, 2], 80% of the cases are discovered in the first two years of life [3]. Therefore, it is not surprising that surgeons may not encounter even a single adult case in their career. Colonic duplication occurs in less than 15% of cases [4–6] with specifically the cecum being the least frequent site, in 4% of cases [6]. Duplications show an intimate anatomic association with or without communication with the alimentary tract, a well-formed smooth muscle layer, and an epithelial lining consisting of some portion of the alimentary tract and share a common blood supply with the adjacent viscera [2, 8–10]. They can be tubular in 14% of the cases or cystic, as in the present case, in 86% of the cases [2, 10].

Differential diagnosis comprises mesenteric cyst, omental cyst, lymphangioma, false diverticula, or Meckel's diverticula [1, 10, 11]. In the present case specifically, the cystic lesion was mistaken for a hydrosalpinx because of the close anatomic relationship between the cecum and the right uterine adnexa even with magnetic resonance imaging. Appendiceal mucocele was also considered as an alternative diagnosis. Symptoms presented by the patient were initially sporadic and nonspecific, which is a usual observation for patients reaching adulthood with ‘silent' cyst [4]. The cystic lesion was stable over nine months. The management was consistent with the presumed and most prevalent conditions; namely, hydrosalpinx or appendiceal mucocele, yet ruled out by diagnostic laparoscopy. A thorough examination, though with limitations of tactile sensation during laparoscopy, did not allow to feel any obvious mass, which was evidently behind the cecum. The cystic nature of the lesion also explains softness during laparoscopic palpation. Because of the young age of the patient and the absence of threatening condition, further investigation was decided with colonoscopy, before embarking on a blind resection. It remains difficult to conclude about the possibility that appendectomy precipitated the ultimate episode of intussusception, as no case being reported since the advent of laparoscopic approach. It became however, evident that the patient was previously presenting with recurrent but spontaneously resolving episode of intussusception.

Intussusception is commonly encountered in pediatric population [12] and is 20 times more frequent as a cause of bowel obstruction in comparison with adult population [13]. Intussusception represents only 1% of intestinal obstruction in adult population [12–14], the ileocolic form of intussusception occurring in about one third of cases [12]. About 70% to 90% of intussusceptions in adults have a well-defined pathological lead point, which is malignant in about 60% of cases [15]. The risk of finding a malignancy increases with the patient's age but remains possible even in young adults [17]. Benign lesions such as adenoma, lipoma, inflammatory polyps, leiomyoma, neurofibroma, and appendiceal tumor may cause ileocolic or colocolic intussusception [12, 14, 15]. In a systematic review and meta-analysis that included 40 retrospective studies with a total of 1229 patients, Hong et al. [17] reported that 29% of the cases were ileocolic intussusception of which 61.7% were due to primary adenocarcinoma. Resection is thus always indicated [17].

Intussusception involving the colon remains very rare encounter in a surgeon's practice. Azar and Berger retrieved only fourteen cases of colonic intussusception in a 30-year period at Massachusetts General Hospital [13], but the ileocolic type was not specifically defined. More recently, Kim found 13 cases (10 ileocolic; 3 ileocecal) during a 10-year period [15]. Shenoy also reported 11 cases of ileocecal intussusception in thirteen years [16]. Not even one case of cecal duplication as a cause of intussusception was mentioned from any of these case series in adults.

Kim et al. first reported in 2014, a case of 19-year-old patient with an ileocolic intussusception from an ileal duplication in an adult [11]. However, the first well-described case involving specifically a cecal duplication cyst leading to intussusception in a 24-year-old patient was published by Al-Shaibi et al. [3]. A second case with a cecal duplication cyst, which occurred in a 63-year-old patient, was recently published, but the authors suggested that the intussusception was triggered by COVID-19 infection [5]. Other cases of intussusception, in patients of age 19 to 35 years, caused by duplications near but not from the cecum have also been published [2, 4, 10, 18].

Even though intestinal duplication has recently gained attention as a potential cause of intussusception in adults [2], occurrence of both cecal duplication and intussusception events at the same time remains very rare [2, 11]. Moreover, even for the pediatric population, only few cases of enteric duplications manifesting as an enterocolic intussusception are reported [11, 19]. It remains difficult to estimate the real incidence of alimentary tract duplications reaching adulthood without complications [2, 4].

## 4. Conclusion

In conclusion, both ileocecal intussusception and cecal duplication are very rarely encountered situations in the adult population. The cecal duplication as the main cause for intussusception, although making sense, should remain an anecdotal incidence. The present case is only the third one to be reported. This case, however, underlines two points. First, a cystic lesion in the right iliac fossa may be a duplication cyst that could be confused with an adnexal cyst or hydrosalpinx or an appendiceal mucocele. Second, should an ileocecal intussusception occur even in a young adult, a resection must be done, irrespective of the underlying cause.
